# Pharmacist-led therapeutic carbohydrate restriction as a treatment strategy for type 2 diabetes: the Pharm-TCR randomized controlled trial protocol

**DOI:** 10.1186/s13063-019-3873-7

**Published:** 2019-12-27

**Authors:** Cody Durrer, Sean McKelvey, Joel Singer, Alan M. Batterham, James D. Johnson, Jay Wortman, Jonathan P. Little

**Affiliations:** 1 School of Health and Exercise Sciences, University of British Columbia, Kelowna, BC V1V 1V7 Canada; 2 Institute for Personalized Therapeutic Nutrition, Vancouver, BC Canada; 3 School of Population and Public Health, University of British Columbia, Vancouver, BC Canada; 4 Centre for Rehabilitation, Exercise and Sports Science, Teesside University, Middlesbrough, UK; 5 Diabetes Research Group, Life Sciences Institute, Faculty of Medicine, University of British Columbia, Vancouver, BC Canada; 6 Faculty of Medicine, University of British Columbia, Vancouver, BC Canada

**Keywords:** Diabetes, Ketogenic diet, Glucose, A1C, Diet, Nutrition, Pharmacy, Insulin

## Abstract

**Background:**

The current treatment paradigm for type 2 diabetes mellitus (T2D) typically involves use of multiple medications to lower glucose levels in hope of reducing long-term complications. However, such treatment does not necessarily address the underlying pathophysiology of the disease and very few patients achieve partial, complete, or prolonged remission of T2D after diagnosis. The therapeutic potential of nutrition has been highlighted recently based on results of clinical trials reporting remission of T2D with targeted dietary approaches. During the initial phase of such interventions that restrict carbohydrates and/or induce rapid weight loss, hypoglycemia presents a notable risk to patients. We therefore hypothesized that delivering very low-carbohydrate, low-calorie therapeutic nutrition through community pharmacies would be an innovative strategy to facilitate lowering of glycated hemoglobin (A1C) while safely reducing the use of glucose-lowering medications in T2D.

**Methods:**

A community-based randomized controlled trial that is pragmatic in nature, following a parallel-group design will be conducted (*N* = 200). Participants will have an equal chance of being randomized to either a pharmacist-led, therapeutic carbohydrate restricted (Pharm-TCR) diet or guideline-based treatment as usual (TAU). Pharm-TCR involves a 12-week very low carbohydrate, calorie-restricted commercial diet plan led by pharmacists and lifestyle coaches with pharmacists responsible for managing medications in collaboration with the participants’ family physicians. Main inclusion criteria are diagnosis of T2D, currently treated with glucose-lowering medications, age 30–75 years, and body mass index ≥ 30. The primary outcome is a binary measure of use of glucose-lowering medication. Secondary outcomes include A1C, anthropometrics and clinical blood markers.

**Discussion:**

There are inherent risks involved if patients with T2D who take glucose-lowering medications follow very low carbohydrate diets. This randomized controlled trial aims to determine whether engaging community pharmacists is a safe and effective way to deliver therapeutic carbohydrate restriction and reduce/eliminate the need for glucose-lowering medications in people with T2D.

**Trial registration:**

ClinicalTrials.gov, NCT03181165. Registered on 8 June 2017.

## Background

Diabetes mellitus affects ~ 10% of the Canadian population, with worldwide estimates that 628 million will have diabetes mellitus by 2045 [1]. Approximately 90% of all cases are type 2 diabetes mellitus (T2D) [1]. T2D contributes to a significant increase in morbidity and mortality due to numerous complications attributed to hyperglycemia, hyperinsulinemia, insulin resistance, and associated metabolic dysfunction [2]. The current treatment paradigm for T2D typically involves use of multiple medications to lower glucose levels in hopes of reducing long-term complications [3]. Specifically, if patients with T2D fail to lower hemoglobin A1C (A1C) through lifestyle modification within 3 months, they are started on a treatment regimen beginning with metformin and progressing with the addition of other antihyperglycemic agents until A1C targets are achieved. These antihyperglycemic agents operate through a variety of mechanisms to lower blood glucose; however, the mortality benefits of different add-on medications appear to be different [4–7]. However, this treatment does not necessarily fix the underlying pathophysiology of the disease and studies indicate that T2D is typically a chronic, progressive disease with only < 2% of patients achieving partial, complete, or prolonged remission of diabetes mellitus after diagnosis [8]. When patients are taking multiple medications, the treatment plan requires involvement and integration of care from not only physicians but also pharmacists. Indeed, it has been demonstrated that patients with T2D have more annual visits to their pharmacist than their primary care physician, placing the pharmacist in a key position for overall management of diabetes mellitus [9].

Lifestyle interventions including diet and exercise are often stated as frontline treatments for T2D, either alone or in combination with medications [10, 11]. In particular, the therapeutic potential of nutritional interventions has been highlighted recently based on results of clinical trials reporting “remission” of T2D with targeted dietary approaches [12, 13]. In a non-randomized trial, a remote continuous care model delivering a very low carbohydrate, high fat, non-calorie-restricted diet was effective at lowering A1C, promoting weight loss, and reducing the use of glucose-lowering medications (including insulin) in patients with T2D [14, 15]. The authors reported T2D “reversal” (defined in this study as A1C below diabetes mellitus thresholds and taking no glucose-lowering medications except for metformin) at both 10 weeks [15] and 1 year [14]. In a cluster randomized controlled trial (RCT) involving primary care practices, Lean et al. [12] showed that a calorie-restricted (~ 850 kcal day) total diet replacement method followed by food reintroduction at 12 weeks was effective at inducing remission of T2D (defined as non-diabetic A1C and taking no glucose-lowering medications) in 46% of patients at 1 year. Remission of T2D in this trial was tied to not only weight loss [12] but also rescue of beta-cell function [16]. It is currently unclear whether a very low carbohydrate (< 50 g/day) diet [17] or a more prescriptive low-calorie approach [12] is superior for T2D remission/reversal and we are unaware of any trials specifically comparing these two therapeutic nutrition options. However, it stands to reason that a combined approach incorporating a diet that is both very low carbohydrate and low calorie may be the most potent for lowering blood glucose, promoting weight loss, and achieving T2D reversal.

During the initial phase of therapeutic nutrition interventions that restrict carbohydrates and/or induce rapid weight loss, hypoglycemia presents a notable risk to patients [18, 19]. Indeed, it has been demonstrated that, even prior to weight loss, low carbohydrate diets result in lower glycemia in people with T2D [20]. While rarely addressed in depth, careful medication management and reduction during this period are essential to prevent hypoglycemia and minimize risk [21, 22]. Pharmacists are well-positioned for this role and are educated in medication management and T2D. Studies exploring integrative care wherein pharmacists play an active role in T2D management have demonstrated positive impacts on T2D outcomes [23]. We therefore hypothesized that the innovative strategy of delivering very low carbohydrate, low-calorie therapeutic nutrition through community pharmacies would facilitate lowering of A1C while safely reducing the use of glucose-lowering medications in T2D, particularly in more complex cases in which individuals are taking multiple medications or insulin. To this end, we designed an RCT to determine if community pharmacist-led therapeutic carbohydrate restriction could eliminate glucose-lowering medications and facilitate T2D reversal/remission.

### Study objectives

The study objectives are:

1. To determine if people with T2D are able to reduce and/or eliminate their need for glucose lowering medications by utilizing a pharmacist-led, therapeutic carbohydrate restricted (Pharm-TCR) diet.
2. To determine if following the Pharm-TCR diet will lead to improvements in body composition, blood glucose control, and cardiometabolic health in people with T2D.
3. To determine if following the Pharm-TCR diet will lead to improvements in quality of life in people with T2D

Data will also be collected for possible future health economics analysis (e.g., medication costs). Trial results will be published.

## Methods/design

**Fig. 1:**
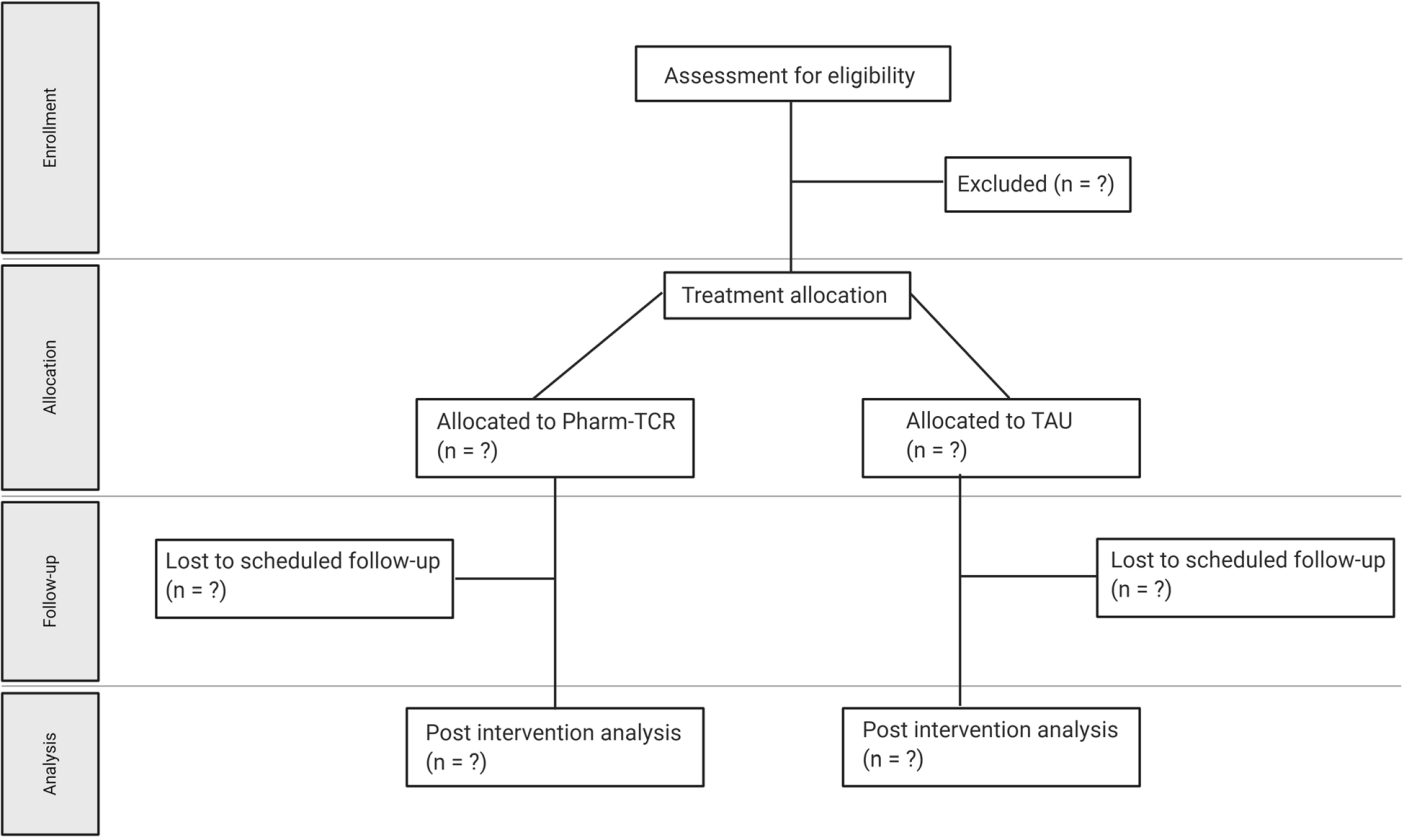
Trial Flow Diagram Pharm-TCR, Pharmacist-led, therapeutic carbohydrate restricted diet; TAU, Treatment-as-usual

A community-based randomized controlled trial that is pragmatic in nature, following a parallel-group design will be conducted. Patients with T2D (*N* = 200) will be recruited from across 12 pharmacy locations in the province of British Columbia, Canada. The pharmacists will use a secure password-protected website maintained by the University of British Columbia (UBC) Centre for Health Evaluation and Outcome Sciences (CHEOS) to implement the random allocation sequence. The secure website provides allocation concealment until the interventions are assigned. The allocation sequence generated was by a researcher from CHEOS using PROC PLAN in SAS 6.4 software. Participants will be stratified by site (pharmacy) and glucose-lowering medications (two or fewer, three or more (or taking exogenous insulin)) and randomized in a 1:1 allocation ratio using permuted block sizes, to either the therapeutic nutrition group or the treatment-as-usual (TAU) group. Due to the nature of the interventions and the involvement required of the pharmacists, they cannot be blinded to final treatment allocations of the participants. The protocol was approved by the University of British Columbia Clinical Research Ethics Board (H16–01539) and the trial was registered on ClinicalTrials.gov (NCT03181165) on 8 June 2017. A flow diagram of the trial is presented in Fig. 1. Additional file 3 contains the SPIRIT 2013 Checklist.

### Primary outcome

The primary outcome is the proportion of patients with T2D who are either using or no longer using glucose-lowering medications after the 12-week study period.

### Secondary outcomes

The secondary outcomes are:

- Hemoglobin A1C (%)
- Percentage dose reduction in glucose-lowering medications (change in daily dose)
- Body mass index (BMI) (kilograms/meters squared) and weight reduction (kilograms)
- Waist circumference (centimeters)
- Body fat percentage (%)
- Health-related quality of life (SF-20)
- Blood lipid profile (total, high-density lipoprotein (HDL) and low-density lipoprotein (LDL) cholesterol, triglycerides), liver function tests (alanine aminotransferase (ALT), aspartate aminotransferase (AST), gamma-glutamyl transpeptidase (GGT)), fasting plasma glucose, pro-insulin, insulin, and C-peptide
- Blood pressure (millimeters of mercury)
- Percentage dose reduction in blood pressure-lowering medications (change in daily dose)

### Participant recruitment

We aim to recruit 200 participants (equal probability of randomization to each arm) using a combination of newspaper advertising, online advertising, recruitment posters posted in pharmacies, and by word of mouth in each respective study site location. Potential participants who respond to the recruitment material will be directed to the nearest study site and invited to attend a screening appointment where the study will be fully explained and discussed. Participants will provide written informed consent and their primary care physician will be informed of their participation prior to any data collection. The pharmacists at each site will obtain consent.

#### Inclusion criteria

The inclusion criteria are:

- Written informed consent
- Men and women aged 30–75 years
- Diagnosed with T2D by a physician
- Using at least one glucose-lowering medication
- BMI ≥ 30 kg·m^2^

#### Exclusion criteria

The exclusion criteria are:

- History of heart attack or coronary artery disease within the previous 2 years
- Any current unstable cardiovascular disorder
- History of liver disease
- History of kidney disease or impaired renal function
- Currently pregnant or lactating, or planning on becoming pregnant within the next 12 months
- Any diagnosed neurological disorder
- History of bariatric surgery
- History of cancer within the previous 5 years
- Dietary restrictions that would inhibit adherence to the intervention diet

### Trial procedures

Eligible participants who enroll in the study will undergo baseline assessment of medications and A1C, fasting glucose, triglycerides, liver ALT, AST, GGT, serum triglycerides, HDL and LDL cholesterol, and high sensitivity (hs)-C-reactive protein (hs-CRP). Weight, height, BMI, body fat percentage (measured by bioelectrical impedance), and waist circumference will be taken as anthropometric measurements, and quality of life will be assessed using the SF-20 questionnaire. Participants’ primary care physicians will be notified of all laboratory results as per typical blood requisition (by fax). Participants will fill out a 3-day diet record to assess habitual food intake and the Godin Leisure-Time Exercise Questionnaire to assess typical physical activity. Participants will also be asked to fill out a questionnaire regarding their ethnicity and socioeconomic status. Following the baseline assessments, participants are randomized to either the Pharm-TCR diet group or the TAU control group. A simplified cost-effectiveness analysis including the cost of medications and cost of the foods and pharmacists’ time during the intervention will be conducted as an exploratory outcome.

### Pharm-TCR intervention (0–12 weeks)

Participants randomized to the Pharm-TCR group will meet with a pharmacist and a lifestyle coach at a community pharmacy. The pharmacist will review all medications with the participant and then develop a care plan, including medication recommendations (Additional file 1), to be shared and approved by the participant’s physician (by fax, using existing standard procedures for any medication adjustments recommended by pharmacists). Any adjustments to care made by the physician will be incorporated into the plan. The lifestyle coach or pharmacist will then educate the participant on the components of the intervention and review the Pharm-TCR food options, which are standardized by using a combination of whole foods and supplemental foods from a commercial weight loss diet (Ideal Protein). This commercial diet plan (provided to the participants free of charge) provides a variety of low-carbohydrate, energy-restricted, adequate-protein meals and snacks that are used in combination with a selected group of meats and a wide range of vegetables in order to create a personalized meal plan for each participant. Participants will not otherwise be remunerated for participating in the trial. The macronutrient composition of the meal plan equates to < 50 g of carbohydrates, ~ 35-45 g of fat, and ~ 110-120 g of protein for a total of approximately 850–1100 kcal per day. Pre-packaged foods are used for two meals and one snack each day, with the third meal being prepared from a select group of lower-fat protein sources (e.g., meat, eggs) and low-carbohydrate vegetables. The use of a commercial weight loss diet plan allows for tight control over the amount of carbohydrates consumed and the overall energy intake. This approach was chosen because the consistency makes medication adjustments more predictable and reproducible for the pharmacist. This is essential as the pharmacists will need to manage medication adjustments in anticipation of, and in response to, the rapid glucose-lowering effects of this specific low-carbohydrate low-calorie dietary intervention, even prior to substantial weight loss. For patients who are taking > 30 units of exogenous insulin per day at the start of the study, a modified TCR meal plan is used, which includes 80–100 g of carbohydrates per day. This is to reduce potential risk of hypoglycemia during the insulin de-escalation phase of the study (details below and in Additional file 1). Once a participant’s insulin dose is < 30 units per day they are transitioned to the regular TCR meal plan incorporating < 50 g of carbohydrate per day with remaining insulin dose being reduced by a further 50% on day 1.

Following the initial visit, participants will be required to report to the pharmacy each week to meet with the lifestyle coach and pharmacist in order to monitor progress, collect intervention foods, and receive counseling on dietary adherence for the week ahead (total of 12 visits). At this time, the pharmacist will review the current status of the care plan with the patient, make adjustments as necessary, and communicate as appropriate with their physician. The care plan is individualized to each participant based on their baseline medication usage and blood glucose measurements using a standardized medication deprescribing framework as outlined in Additional file 1. Participants’ medication usage, weight, height, waist circumference, body fat percentage, blood pressure, and capillary ketone levels are also recorded during these visits and a 3-day diet record is filled out again on week 6. On the final visit, participants are assessed for the same blood, anthropometric, quality of life, and habitual physical activity measures that were collected at baseline.

### Treatment-as-usual control (0–12 weeks)

Participants randomized to the TAU group will undergo a medication review by the pharmacist, will be provided with standard medication advice by their pharmacist, and will be given information pamphlets on diet and lifestyle conforming with 2013 Diabetes Canada (formerly the Canadian Diabetes Association) Clinical Practice Guidelines. This information is evidence based and recommends participants to follow a low-fat, low glycemic-index diet and to engage in 150 min of moderate activity per week. Standard clinical practice recommends an A1C test and potential medication adjustments every 3 months (typically an increase in dose or number of glucose-lowering drugs if A1C does not reach below the clinical target of < 7.0%) and therefore the treatment period of 12 weeks was chosen.

Three-day food records will be collected at baseline, week 6, and week 12 to assess the energy and macronutrient content of the foods consumed. Following week 12, TAU participants will return to the pharmacy for a final visit where they are assessed again for all baseline blood, anthropometric, quality of life, and habitual physical activity measures.

In order to help retain TAU-group participants for follow-up testing they will be offered participation in the Pharm-TCR intervention after the TAU period has completed. If a TAU-group participant chooses to try the dietary intervention following completion of the TAU period, the participant is considered to have finished the trial and as such, no study data will be collected from them during this time period.

### Patient safety

During the first 2–4 weeks of the program (when most medication adjustments are made), participants communicate frequently with their pharmacist and lifestyle coach via phone call and/or text messaging and take finger stick blood glucose measurements four times per day (a fasting measurement upon waking, and before lunch, dinner, and bedtime). This allows for feedback and assessment of the medication reductions and helps to reduce any potential risk of hypoglycemia. Participants are instructed on steps to take if blood glucose is < 3.9 mM and/or they have symptoms of hypoglycemia; the steps include consumption of an energy bar containing 15 g of carbohydrates and, if severe or if symptoms do not resolve, reporting to their doctor or emergency room. All observations/results that may pose a risk to health will be discussed with the study team and all adverse events will be recorded. Pharmacists at each site will be closely engaged with participants due to the nature of the intervention. Any safety concerns will be brought to the attention of the study physician, Dr. Jay Wortman and the lead pharmacist (Mr. Sean McKelvey). A safety committee of two independent physicians with experience in low-carbohydrate diets and T2D will review any adverse events and provide clinical insight into their etiology. These two independent physicians can request stoppage or changes to the ongoing study. The Principal Investigator (PI) (Dr. J. Little) will meet with Dr Wortman, Mr. McKelvey, and the two independent physicians every 6 months or as often as needed, to review any safety issues or data collection issues. If there are any serious adverse events, the independent physicians or study physician can recommend that any/all participants discontinue participation in the trial. Participants may also choose to discontinue at any time. Pharmacists at each site will have access to the data as they are collected. They will fax or email any data, with no personal identifiers, to the PI or study email address at the UBC. Only study investigators will have access to the faxed or emailed data. Any personally identifying data collected by the pharmacists will be de-identified prior to being sent to the research team.

### Protocol delivery fidelity

A standard training protocol has been developed to minimize variability and maintain the intervention protocol across all pharmacies. Training videos followed by visits in person by the study coordinator will be used to explain the study design, aims, and protocol to the study personnel at each pharmacy. The study coordinator will remain easily accessible to the pharmacy personnel via email and phone throughout the study. The head study pharmacist teaches the medication adjustment framework to the lead pharmacist from each study site, by in-person workshops that include both a lecture portion and multiple case studies. The head study pharmacist will also be available to the site pharmacists via email and phone throughout the study.

### Measurements

The measurements taken at each stage of the study are detailed in the Additional file 2. Height will be measured to the nearest millimeter, using a stadiometer (Seca, model 700, Germany). Body weight will be measured to the nearest 100 g (Tanita, model DC-430 U, IL, USA) with the participant wearing light clothing without shoes. Waist circumference is assessed by measuring the distance around the waist at the top of the iliac crest. Blood pressure will be measured using the PharmaSmart Model PS-2000C (BC, Canada) after the participant has been seated, at rest, with legs uncrossed for at least 5 minutes. Body fat percentage will be assessed by bioelectrical impedance analysis (Tanita, model DC-430 U, IL, USA). Briefly, participants stand on the scale with soles and heels of both of their feet in contact with the electrodes and the participants’ sex, height, and weight are used in combination with the measured resistance to calculate body fat percentage. Tanita has validated this method in people with T2D against dual x-ray absorptiometry (r = 0.89, *p* < 0.0001) [24].

Participants in the Pharm-TCR group will have their capillary ketone levels measured at each weekly visit using a Freestyle Precision Neo device (Abbott Laboratories, CHI, IL, USA). Clinical laboratory requisitions will be given to the participants for blood to be collected at a local clinical laboratory (LifeLabs or Valley Medical Labs) at baseline and post Pharm-TCR intervention or TAU control, to measure A1C, fasting glucose, triglycerides, ALT, AST, GGT, serum triglycerides, HDL and LDL cholesterol, and hs-CRP. A subsample of participants will have fasting C-peptide, insulin, and proinsulin measured to estimate homeostasis model assessment of insulin resistance (HOMA-IR) and beta-cell function based on HOMA-B and C-peptide–proinsulin ratio. This subsample analysis of the plasma samples collected by the clinical laboratory will be performed by the research team at a university laboratory.

### Statistical analysis

Data will be analyzed on an intention-to-treat basis at the 12-week (post-testing) timepoint. The primary outcome measure is binary - using or not using glucose-lowering medication at 12 weeks - and data will be analyzed by logistic regression, with the stratified allocation factors included as fixed effects together with trial arm and sex. We will derive the odds ratio for use of glucose-lowering medication, and odds will be converted to predicted probabilities (risk differences). Participants who fail to complete the intervention or are lost to follow up from the Pharm-TCR group will be retained in the analyses and will be considered as not achieving the primary outcome. The secondary outcome measures of anthropometrics, blood pressure, medication dose changes, quality of life, and all blood measures will be analyzed using constrained baseline longitudinal data analysis via a linear mixed model [25]. This approach provides a principled method for addressing missing data, including missing baseline data as baseline values are part of the response vector in this model. In addition, study site will be included as a random effect in the linear mixed models to account for site variability in the outcome measures [26]. Model specification will be examined by visual inspection of residuals plots, with appropriate data transformations used if indicated. For the primary endpoint we shall report the odds ratios and predicted probabilities (risk differences) with 95% confidence intervals. Secondary continuous outcomes will be reported as mean differences with 95% confidence intervals. Whenever possible, data analysis will be performed blinded to the allocations.

It is not anticipated that any participants in the TAU group will discontinue glucose-lowering medications; however, to be conservative, the sample size was calculated based on an expected proportion of medication use of 80% in the TAU group and a 20% difference in the proportion of participants on glucose-lowering medications at 12 weeks in the Pharm-TCR group. A sample size of 100 per group provides 80% power to detect a 20% difference in the proportion of patients on glucose-lowering medications, accounting for 20% attrition with a two-sided *P* value of 0.05 (PASS 14 Power Analysis and Sample Size Software, 2015. NCSS, LLC. Kaysville, Utah, USA; ncss.com/software/pass).

## Discussion

The International Diabetes Federation (IDF) estimates the total healthcare expenditure for people with diabetes mellitus to be ~ US$747 billion in 2017, a burden that is expected to grow by at least 7% by the year 2045. A substantial portion of these healthcare costs are attributed to the cost of diabetes medications; in Canada, the direct costs related to drugs for treating T2D were ~CA$84 million in 2010 [27]. Currently, the usual treatment paradigm for a patient newly diagnosed with T2D is to begin drug therapy with the goal of achieving acceptable blood glucose levels [3]. This approach has been demonstrated to lead to extremely low levels of T2D remission [8] and more commonly will lead to steady progression of the disease and eventually multi-drug therapy in order to maintain target blood glucose levels. Reversal and remission of T2D has now been shown to be possible through substantial weight loss following bariatric surgery [28] or by following a calorie-restricted diet [12]. In addition, there is evidence that very low carbohydrate diets are also effective in managing and possibly reversing the progression of T2D [14, 29]. While these studies are encouraging, translation of these findings into the real-world setting must be approached with considerable care due to risk of hypoglycemia when patients with T2D taking glucose-lowering medications adopt therapeutic nutrition. A key principle in this study is the careful monitoring and systematic deprescription (Additional file 1) of medications by trained pharmacists to address this safety concern.

In order to assess the performance of the Pharm-TCR intervention, primary and secondary outcomes will be compared to the standard-care treatment for patients with T2D. Typically a person with T2D is placed on antihyperglycemic pharmacotherapy if they are above their individualized A1C target with additional agents and or dose adjustments added as needed to achieve the target A1C [30]. By using this comparison, the trial is designed to be as realistic as possible so that the findings can be assessed in the context of real-world T2D management and allow for greater potential to be translated into routine health care.

A randomized controlled trial design was chosen to protect against potential bias arising from individual study sites such as location, socioeconomic status of the study site area, and possible differences in pharmacy care. Although this meant that a given study site would have to deliver different patient management to randomly allocated patients, the “hands-off” nature of the TAU control and the limited interaction involved does not allow the opportunity for the pharmacist to negatively bias outcomes in the TAU control-group participants. Nonetheless, the Pharm-TCR intervention is more intense and participants necessarily receive more attention in the chosen RCT design comparing the intervention to TAU as opposed to an alternate dietary intervention or weekly counseling control group.

A key principle behind this study is the emphasis on safe deprescription of medications during the intervention period with a focus on controlled glycemia. Furthermore, the aim is to achieve this by using an underutilized aspect of a care network for patients with T2D, the pharmacist, who is well-positioned and educated for such a task. While the implementation of a diet intervention by pharmacists is not a conventional strategy, we utilized a standardized, commercial weight-loss diet program such that pharmacists did not need to be experts in nutrition. This allows pharmacists to use their unique position of frequent patient interaction and extensive knowledge of medications to safely and successfully administer the intervention and treatment plan. Timely medication adjustments are essential, as blood glucose levels can be reduced even in the absence of weight loss when following low-carbohydrate diets [20]. In addition, all medication adjustments must be approved by the participants’ physicians, which provides another layer of safety to the deprescription procedure and leverages established methods of communication in place for pharmacists and physicians in Canada. For these reasons, pharmacists are an innovative, yet natural, choice to safely and effectively implement such an intervention.

The choice for the primary outcome to be a dichotomous measure of glucose-lowering medication use was made in an effort to select a clinically relevant outcome. Participants that are recruited for this study are expected to have wide variability in prescribed types and doses of glucose-lowering medications. Therefore, determining a clinically meaningful dose reduction that would apply to all possible medication scenarios is not feasible. However, if a T2D treatment is able to eliminate the need for glucose-lowering medications entirely, in the absence of significant worsening in glycemia, there is no doubt it can be considered clinically relevant. The secondary outcomes of A1C and fasting glucose will test whether the very low carbohydrate and calorie-restricted dietary approach can benefit glucose control, and secondary anthropometric outcomes (weight, waist circumference, and body fat percentage) will also support the primary outcome, as substantial weight loss in patients with T2D is associated with T2D remission [12]. With careful documentation of glucose-lowering medication use and measures of glucose control, it will also be possible to report on exploratory outcomes of short-term T2D “reversal” using various definitions that have been suggested in the literature, including (1) no glucose-lowering medications, A1C below diabetes mellitus threshold; (2) no glucose-lowering medications, A1C and fasting glucose below diabetes mellitus thresholds; (3) no glucose-lowering medications, A1C and/or fasting glucose in the true normoglycemic range; and (4) A1C below diabetes mellitus threshold and taking only metformin [14, 31]. Though sex will be included as a fixed effect in the logistic regression and linear mixed effects models, a limitation of this study is the inability to determine gender differences in the effectiveness of the intervention. Finally, while a novel aspect of this study is combination of the independently successful strategies of calorie-restriction and low-carbohydrate approach to treating T2D, a limitation of this design is the inability to separate the effects of the two strategies.

## Conclusion

This study will provide evidence to inform and aid in future T2D management using pharmacist-led therapeutic carbohydrate restriction. It provides insight into implementing dietary strategies that are increasingly being recognized as beneficial for people with T2D with a focus on safe and efficient implementation into care models. The protocol is optimized to deploy pharmacists, who are well-educated in medication management and ideally positioned to carry out this T2D treatment strategy in the community. This trial will determine if community pharmacists can deliver a very low-carbohydrate, calorie-restricted diet to eliminate the need for glucose-lowering medication without causing an increase in A1C.

## Supplementary information

**Additional file 1.** Deprescription plan.

**Additional file 2.** Schedule of assessments.

**Additional file 3.** SPIRIT 2013 Checklist: Recommended items to address in a clinical trial protocol and related documents.

## Data Availability

There is no plan to publicly make individual participant data freely available; however, these data will be made available upon request.
