# Supplementary material for: Pharmacist-led therapeutic carbohydrate restriction as a treatment strategy for type 2 diabetes: the Pharm-TCR randomized controlled trial protocol
Source: Trials. 2019 Dec 27;20:781. doi: 10.1186/s13063-019-3873-7 (PMC6935079; doi:10.1186/s13063-019-3873-7)
Supplement: Supplementary file 1 — Additional file 1. Deprescription plan. [file 13063_2019_3873_MOESM1_ESM.docx]

| **Additional File 1**: Deprescription Plan | | | | | |
| --- | --- | --- | --- | --- | --- |
| Category^a^ | Medications | Day 1 Change^b^ | Adjustments | DG Monitoring | Notes |
| High Risk | Insulin | Reduce by 50% LCKD  Reduce by 25-40% LC | Reduce 10-25% if BG<7 | 4x/day ac & hs | For over 30 units start on LC (net >80g/day)  D/C if <=10units/day and BG<7 |
|  | Sulfonylureas  Meglitinides | Reduce by 50% | D/C day 10 to 14 or before if BG<6mmol/L | 2x/day + |  |
|  | SGLT2i | D/C Day 1 | Use LC >80g/d if not D/C. Monitor BG and D/C if BG<6mmol/L | 2x/day + | Increased risk of Euglycemic DKA (EDKA) while on TCR |
| Moderate Risk | TZD & DPP4i | Reduce by 50% | D/C day 10 to 14 or before if BG<6mmol/L | 2x/day |  |
|  | GLP 1 analogs | Reduce by 1/3 to 1/2 | Consider reduction or D/C if BG consistently within range | 2x/day |  |
| Low Risk | Metformin | Consider 50% reduction if monotherapy  Changes not required | Consider D/C if BG consistently within range | Initially 2x/day then PRN | Monitor for GI side effects |

^a^ Risk categories based on clinical experience using therapeutic carbohydrate restriction (TCR)

^b^ Pharmacists can only recommend changes. Physician must approve before implemented
