# Supplementary material for: Pharmacist-led therapeutic carbohydrate restriction as a treatment strategy for type 2 diabetes: the Pharm-TCR randomized controlled trial protocol
Source: Trials. 2019 Dec 27;20:781. doi: 10.1186/s13063-019-3873-7 (PMC6935079; doi:10.1186/s13063-019-3873-7)
Supplement: Supplementary file 2 — Additional file 2. Schedule of assessments. [file 13063_2019_3873_MOESM2_ESM.docx]

| **Additional File 2**: Schedule of assessments | | |  |  |  |  |  |  |  |  |  |  |  |  |  |  |
| --- | --- | --- | --- | --- | --- | --- | --- | --- | --- | --- | --- | --- | --- | --- | --- | --- |
| **Visit** | 1 | 2 | | | 3 | 4 | 5 | 6 | 7 | 8 | 9 | 10 | 11 | 12 | 13 | 14 |
| **Week** | 0 | 0 | | | 1 | 2 | 3 | 4 | 5 | 6 | 7 | 8 | 9 | 10 | 11 | 12 |
| **Study Procedure** |  |  | | |  |  |  |  |  |  |  |  |  |  |  |  |
| Review & discuss study participation | **✔** |  | | |  |  |  |  |  |  |  |  |  |  |  |  |
| Review inclusion & exclusion criteria | **✔** |  | | |  |  |  |  |  |  |  |  |  |  |  |  |
| Obtain informed consent | **✔** |  | | |  |  |  |  |  |  |  |  |  |  |  |  |
| Medication Review | **✔** | **✔^a^** | | | **✔^a^** | **✔^a^** | **✔^a^** | **✔^a^** | **✔^a^** | **✔^a^** | **✔^a^** | **✔^a^** | **✔^a^** | **✔^a^** | **✔^a^** | **✔** |
| A1C | **✔** |  | | |  |  |  |  |  |  |  |  |  |  |  | **✔** |
| Fasting Glucose | **✔** |  | | |  |  |  |  |  |  |  |  |  |  |  | **✔** |
| Liver enzymes | **✔** |  | | |  |  |  |  |  |  |  |  |  |  |  | **✔** |
| Cholesterol | **✔** |  | | |  |  |  |  |  |  |  |  |  |  |  | **✔** |
| Triglycerides | **✔** |  | | |  |  |  |  |  |  |  |  |  |  |  | **✔** |
| CRP | **✔** |  | | |  |  |  |  |  |  |  |  |  |  |  | **✔** |
| Beta-cell function assessment | **✔^b^** |  | | |  |  |  |  |  |  |  |  |  |  |  | **✔^b^** |
| Height | **✔** | **✔^a^** | | | **✔^a^** | **✔^a^** | **✔^a^** | **✔^a^** | **✔^a^** | **✔^a^** | **✔^a^** | **✔^a^** | **✔^a^** | **✔^a^** | **✔^a^** | **✔** |
| Weight | **✔** | **✔^a^** | | | **✔^a^** | **✔^a^** | **✔^a^** | **✔^a^** | **✔^a^** | **✔^a^** | **✔^a^** | **✔^a^** | **✔^a^** | **✔^a^** | **✔^a^** | **✔** |
| Waist circumference | **✔** | **✔^a^** | | | **✔^a^** | **✔^a^** | **✔^a^** | **✔^a^** | **✔^a^** | **✔^a^** | **✔^a^** | **✔^a^** | **✔^a^** | **✔^a^** | **✔^a^** | **✔** |
| Body Fat % | **✔** | **✔^a^** | | | **✔^a^** | **✔^a^** | **✔^a^** | **✔^a^** | **✔^a^** | **✔^a^** | **✔^a^** | **✔^a^** | **✔^a^** | **✔^a^** | **✔^a^** | **✔** |
| Capillary Ketones |  | **✔^a^** | | | **✔^a^** | **✔^a^** | **✔^a^** | **✔^a^** | **✔^a^** | **✔^a^** | **✔^a^** | **✔^a^** | **✔^a^** | **✔^a^** | **✔^a^** | **✔^a^** |
| Blood Pressure | **✔** | **✔^a^** | | | **✔^a^** | **✔^a^** | **✔^a^** | **✔^a^** | **✔^a^** | **✔^a^** | **✔^a^** | **✔^a^** | **✔^a^** | **✔^a^** | **✔^a^** | **✔** |
| Health-related quality of life (SF-20) | **✔** |  | | |  |  |  |  |  |  |  |  |  |  |  | **✔** |
| 3-day diet analysis | **✔** |  | | |  |  |  |  |  | **✔** |  |  |  |  |  | **✔** |
| Physical activity | **✔** |  | | |  |  |  |  |  |  |  |  |  |  |  | **✔** |
| Demographics | **✔** |  | | |  |  |  |  |  |  |  |  |  |  |  |  |

^a^ Pharm-TCR participants only

^b^ Sub-sample of participants
